# Biodistribution and Elimination Study of Fluorine-18 Labeled N^ε^-Carboxymethyl-Lysine following Intragastric and Intravenous Administration

**DOI:** 10.1371/journal.pone.0057897

**Published:** 2013-03-07

**Authors:** Hongzeng Xu, Zhongqun Wang, Yan Wang, Shengda Hu, Naifeng Liu

**Affiliations:** 1 Department of Cardiology, Zhongda Hospital, Southeast University, Nanjing, China; 2 Jiangsu Institute of Nuclear Medicine, Wuxi, China; University of Ulster, United Kingdom

## Abstract

**Background:**

N^ε^-carboxymethyl-lysine (CML) is a major advanced glycation end-product (AGEs) widely found in foods. The aim of our study was to evaluate how exogenous CML-peptide is dynamically absorbed from the gastrointestinal tract and eliminated by renal tubular secretion using microPET imaging.

**Methods:**

The present study consisted of three investigations. In study I, we synthesized the imaging tracer ^18^F-CML by reacting N-succinimidyl 4-^18^F-fluorobenzoate (^18^F-SFB) with CML. In study II, the biological activity of ^18^F-CML was evaluated in RAW264.7 cells and HepG2 cells. In study III, the biodistribution and elimination of AGEs in ICR mice were studied in vivo following tail vein injection and intragastric administration of ^18^F-CML.

**Result:**

The formation of ^18^F-CML was confirmed by comparing its retention time with the corresponding reference compound ^19^F-CML. The radiochemical purity (RCP) of ^18^F-CML was >95%, and it showed a stable character in vitro and in vivo. Uptake of ^18^F-CML by RAW264.7 cells and HepG2 cells could be inhibited by unmodified CML. ^18^F-CML was quickly distributed via the blood, and it was rapidly excreted through the kidneys 20 min after tail vein injection. However, ^18^F-CML was only slightly absorbed following intragastric administration. After administration of ^18^F-CML via a stomach tube, the radioactivity was completely localized in the stomach for the first 15 min. At 150 min post intragastric administration, intense accumulation of radioactivity in the intestines was still observed.

**Conclusions:**

PET technology is a powerful tool for the in vivo analysis of the gastrointestinal absorption of orally administered drugs. ^18^F-CML is hardly absorbed by the gastrointestinal tract. It is rapidly distributed and eliminated from blood following intravenous administration. Thus, it may not be harmful to healthy bodies. Our study showed the feasibility of noninvasively imaging ^18^F-labeled AGEs and was the first to describe CML-peptide gastrointestinal absorption by means of PET.

## Introduction

Advanced glycation end-products (AGEs) are a heterogeneous group of compounds that are formed when reducing sugar reacts in a non-enzymatically with amino acids in proteins and other macromolecules [1]. The accumulation of AGEs in vivo is recognized as one of the risk factors that contributes to various long-term complications of diabetes, such as atherosclerosis [2], [3] and renal failure [4], [5], [6]. There are two major sources contributing to the total pool of AGEs in the body: exogenous AGEs (ingested in food) and endogenous AGEs (formed in the body) [4]. Although the effects of distinct ingredients on AGEs in biologic systems have been studied over the past 40 years, the findings have been inconsistent. Some studies have demonstrated that AGE-rich diets can lead to AGEs deposition in vivo after gastrointestinal absorption or absorption through the circulatory system [7], [8]. However, other studies have shown that exogenous AGEs are not absorbed by the gastrointestinal tract and are not toxic to human health [9], [10], [11]. These findings indicate that the different biodistributions of AGEs may be related to the different functional effects of AGEs [10], [12]. It is still not clear whether elevated exposure to AGEs contributes to AGE deposition in tissues. Therefore, characterising the biodistribution and elimination of AGEs following intravenous and intragastric administration is important.

Molecular imaging is an emerging technology that allows the visualization of interactions between molecular probes and biological targets [13]. Positron emission tomography (PET), as a major molecular imaging tool, provides a reliable non-invasive measurement technique that enables quantitative analysis of the pharmacokinetics of biomolecules in vivo [14]. The potential of PET strongly depends on the availability of suitable radiotracers, compounds that are labeled with short-lived positron tracers. The PET radionuclides that are most widely used usually include ^11^C (t_1/2_ = 20.39 min), ^13^N (t_1/2_ = 9.97 min), and ^15^O (t_1/2_ = 2 min). Although carbon, nitrogen and oxygen are the main constituents in most important molecules with biological activity and their isotopes have very small kinetic isotope effects, it is difficult to obtain total recovery of the radioactivity from the whole organism due to their short radioactive half-lives [15]. Therefore, positron emitting fluorine-18 (^18^F)-labelled compounds were synthesized, which allowed the measurement the radioactivity distribution time profile and the radioactivity concentration in biological samples without destruction of the tissues and unaffected by the chemical composition of the studied samples. Importantly, ^18^F is easy to produce, has favourable physical properties such as a longer half-life (109.8 min) and a lower energy (0.64 MeV) [16], and allows high resolution PET imaging. However, radiochemistry with ^18^F presents special challenges, and direct incorporation of ^18^F at a high specific radioactivity into peptides and proteins is hampered by the harsh conditions of [^18^F]-labeling reactions [17]. To overcome this obstacle, peptide and protein labeling with ^18^F must be accomplished with prosthetic groups. Among these prosthetic groups, the acylation agent N-succinimidyl 4-[^18^F]fluorobenzoate (^18^F-SFB) is certainly the most commonly used [18], [19], and this approach has achieved ^18^F incorporation into a small organic molecule that is capable of being linked to peptides, proteins, oligonucleotides and antibodies under mild conditions [16]. Therefore, in the present study ^18^F-SFB was used as an ^18^F-labeling reagent.

Among the different AGE epitopes, in vitro experiments have demonstrated that N^ε^-carboxymethyl-lysine (CML) represents the major AGE structure both quantitatively and pathophysiologically [20], [21]. CML exists in two forms: free (non-cross-linking) and bound to lysine residues within peptides and proteins (cross-linking) [22]. Establishing a selective and sensitive method to visualise the physiologic, metabolic and molecular biodistribution of CML-peptides in vivo is needed. PET measurements can be performed after any route of drug administration, such as intravenous, inhalation or oral. To date, in the field of AGE molecular imaging, only a few studies have been performed following intravenous administration [23], [24]. Additionally, the amount of ingested protein-bound amino acid derivative AGEs due to the Maillard reaction per day is 100 to 300 µmol (25 to 75 mg), and they are primarily composed of CML and pyrraline peptides [25]. However, how the gastrointestinal tract deals with this enormous load of non-physiological amino acids remains unclear. Studies of the in vivo metabolism and biodistribution of AGEs following gastrointestinal administration are clearly of great interest.

In the current study, we chose ^18^F-SFB as an agent to modify CML at its free amino group, resulting in the corresponding 4-[^18^F]fluorobenzoylated derivatives, which should be a useful model for CML-containing dipeptides that could potentially result from the digestion of glycated food proteins [23]. We used a suitable radiolabeling method to characterize the ^18^F-CML derivatives using high-performance liquid chromatography (HPLC) and electrospray ionization mass spectrometry. Furthermore, the stability of ^18^F-CML was investigated in vitro and in vivo. Another objective of this study was to compare the elimination and biodistribution of CML following intragastric and intravenous administration.

## Materials and Methods

### Materials

All of the reagents purchased were analytical grade and used as received without further purification, unless otherwise stated. CML was purchased from PolyPeptide, Inc. (Strasbourg, France). ^19^F-SFB and ortho-phtalaldehyde (OPA) were obtained from Sigma-Aldrich (St. Louis, Missouri, USA).

Unless otherwise stated, the level of radioactivity was determined using a CURIEMENTOR 3 detector system (German PTW Co.). High-performance liquid chromatography (HPLC) analysis was performed using an HPLC system (Waters 1525 binary pump and a Waters 2487 dual-λ absorbance detector). A microPET system was used for in vivo imaging (Inveon, Siemens Co. German). All of the instruments were supplied by the Jiangsu Institute of Nuclear Medicine unless otherwise specified.

For the uptake study, two cell lines were used. The RAW264.7 macrophage cell line and HepG2 cell line were purchased from the American Type Culture Collection (ATCC). For in vivo imaging studies, ICR mice were purchased from the Shanghai SLAC Laboratory Animal Center.

All of the animal procedures and experimental protocols were approved by the Southeast University Institutional Animal Care and Use Committee, and the number of animals used and their suffering were minimised as much as possible.

### ^19^F-CML standard products synthesis

**Figure 1:**
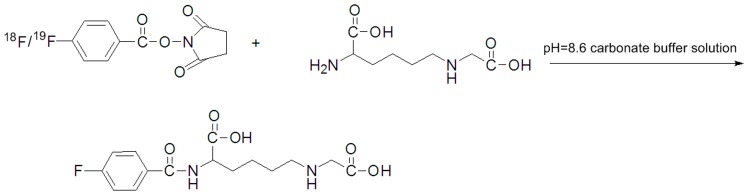
Scheme of ^18^F-CML/^19^F-CML synthesis.

In order to characterize ^18^F-CML on HPLC, the cold compound, ^19^F-CML, was synthetized by conjugating of ^19^F-SFB with CML as a reference standard (Figure 1). ^19^F-SFB (288 µg, 1.2 µmol) in acetonitrile (48 µl) was added to a solution of CML (63 µg, 0.31 µmol) in a carbonate buffer solution (18 µl, pH = 8.4). The reaction mixture was stirred at 65°C in an oil bath for 30 min. The mixture was then purified by semi-preparative HPLC. The fractions were collected and analysed by mass spectrometry. To identify the ^19^F-CML, the molecular weight of each sample was measured on an Autoflex-TOF/TOF mass spectrometer (Bruker Daltonics). The mean molecular mass (m/z) was calculated from multiple independent measurements.

### Synthesis of N-succinimidyl 4-[^18^F]fluorobenzoate (^18^F-SFB)

**Figure 2:**
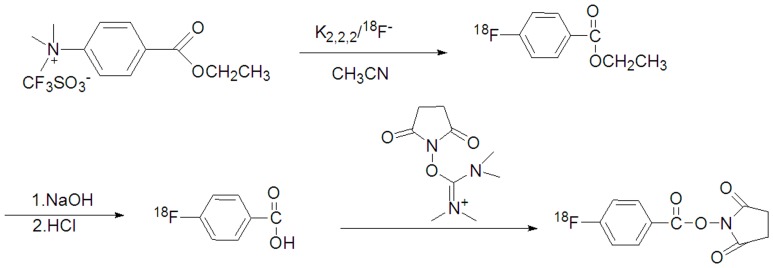
Scheme of ^18^F-SFB synthesis.

Figure 2 shows the reaction scheme of ^18^F-SFB. ^18^F-F^−^ was produced by the ^18^O (p,n) ^18^F reaction in 95% enriched [^18^O]H_2_O using a Cypris HM-7 cyclotron (Sumitomo Heavy Industry). The ^18^F-F^−^ in 1.5 mL of aqueous K_2_CO_3_ (0.02 mmol/L) was transferred into a 5 mL mini-vial (Alltech Co. USA) containing CH_3_CN (0.4 mL)/Kryptofix_222_ (17.72mg). The ^18^F-F^−^ was dried at 100°C for 15 min to remove the H_2_O and CH_3_CN.

The residue was added to a solution of 10 mg of ethyl 4-trimethylammonium triflate benzoate in 0.2 mL of anhydrous CH_3_CN. The reaction mixture was sealed and heated at 80°C for 10 min. The hydrolysis step was performed by adding 0.5 mL of 0.5 mol/L NaOH followed by heating at 90°C for 5 min. After being cooled for 2 min, 0.7 mL of 1 mol/L HCl was added to neutralize the reaction mixture.

The mixture was then diluted to a final volume of 15 mL with distilled water and loaded onto a Waters Sep-Pak cartridge that was activated by passing 30 mL of CH_3_CN and 30 mL of distilled water through the device in advance.

The loaded cartridge was washed with 2 mL of 0.01 mol/L HCl and blown dry for 2 min with a nitrogen stream. The radioactive fraction containing 4-[^18^F]fluorobenzoic acid was eluted with 3 mL of CH_3_CN.

The above radioactive fraction was treated with 20 µL of a 10% aqueous (CH_3_)_4_NOH solution. The reaction mixture was dried in a nitrogen stream at 100°C followed by the addition and evaporation of 1.5 mL of anhydrous CH_3_CN. A solution of O-(N-succinimidyl)-N,N,N′,N′-tetramethyluronium tetrafluoroborate (12 mg) in 0.25 mL of CH_3_CN was added, and the reaction mixture was heated at 80°C for 5 min.

The mixture was concentrated to approximately 0.2 mL, cooled, and diluted with 3 mL of 5% aqueous acetic acid. HPLC semipreparative purification for this mixture was completed on a YMC J'Sphere ODS-H80 column using a mobile phase of CH_3_CN/H_2_O (55%/45%) at a flow rate of 1.0 mL/min. The retention time for ^18^F-SFB was 9.5 min. The ^18^F-SFB fraction was collected, diluted with 6 mL of distilled water, and loaded onto a Sep-Pak C^18^ cartridge. The cartridge was blown dry with nitrogen and eluted with 20.0 µL of CH_3_CN. The CH_3_CN solution of ^18^F-SFB was flowed into the tubing and evaporated to dryness under a gentle nitrogen stream at room temperature.

### ^18^F-CML synthesis

CML was labelled with ^18^F through conjugation coupling with N-succinimidyl 4-[^18^F]fluorobenzoate (^18^F-SFB) (Figure 1). ^18^F-SFB was dissolved in acetonitrile (1 mL) and added to CML (1 mg, 0.3 µmol) dissolved in a carbonate buffer solution (1 mL, pH = 8.4). The reaction was continued for 30 min at 65°C until most of the SFB had reacted according to radio-TLC (acetonitrile 95%). Final purification was accomplished using C18 reversed-phase chromatography (the detection mode was set for radioactivity and the ultraviolet (UV) absorbance was read at a wavelength of 254 nm). HPLC fractions containing radioactivity were combined and evaporated with a stream of argon to remove the acetonitrile.

### ^18^F-CML in vitro stability

The in vitro stability of ^18^F-CML was studied by storing the freshly prepared complex at room temperature (26±2°C) for a period of 6 h. The RCP was evaluated every 0.5 h using paper chromatography to determine its in vitro stability.

### ^18^F-CML in vivo stability

The in vivo stability of the ^18^F-CML in mice was determined using arterial blood and urine samples. Male ICR mice (n = 3) were anaesthetised with 2% vaporized isoflurane and injected with a dose of approximately 3.7 MBq of ^18^F-CML in 250 µL of normal saline via the tail vein. The mice were sacrificed at 15, 60, and 120 min after injection respectively. Blood samples were removed immediately and centrifuged at 12,000 rpm for 2 min at 4°C to separate the plasma. The urinary bladder was carefully excised, and the urine was collected from the bladder. The plasma (30 µL) and an aliquot of the urine sample (20 µL) was injected into an HPLC column to analyze the in vivo stability of ^18^F-CML in the mice.

The HPLC analysis method was based on using trichloroacetic acid to precipitation plasma proteins, which were subsequently separated by reversed-phase column liquid chromatography using a 150 mm×4.6 mm C18 column with a mobile phase of 25 mM acetonitrile and pure water (55%/45%) and detected based on UV absorbance at 254 nm. Radioactivity signal was detected by a radiochromatography detector (PTW, Freiburg, Germany). The ratio of unchanged ^18^F-CML to total radioactivity on the HPLC chromatogram was calculated and expressed as a percentage.

### ^18^F-CML bioactivity

To compare the different bioactivities of CML and ^18^F-CML, two cell lines were evaluated for their capacity to take up native CML and the fluorobenzoylated derivative ^18^F-CML. RAW264.7 macrophages and HepG2 liver cells were cultured in DMEM/LOWGLUCOSE, supplemented with 10% fetal bovine serum (FBS), 4.0 mmol/L L-glutamine, 110 mg/L sodium pyruvate, 100 U/mL penicillin, and 100 mg/L streptomycin at 37°C and 5% CO_2_.

For ^18^F-CML cell uptake studies, RAW264.7 cells and HepG2 cells were seeded into 24-well plates at a density of 4×10^5^ cells per well. Radio-tracer ^18^F-CML (0.5 µCi/well, 3 µmol/mL) was incubated in a 37°C water bath for 15, 30, 60, and 120 min. At each time point the cells were washed 3 times with phosphate-buffered saline (PBS). The supernatant, washing-up liquid (unbound activity) and the pellet (cell-bound activity) were transferred to new tubes. Triplicates of the counting tubes containing supernatant and cell pellet were counted individually using a gamma counter for 1 min. For the blocking experiment, the uptake was carried out in the presence of the non-radioactive CML(10 µmol/mL) and processed similarly as described above. The uptake (%) of radio-tracer into the cell was determined using a gamma counter according to the following equation:
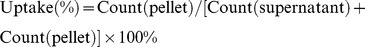



For CML cell uptake ratio studies, unaltered CML (10 µmol/mL) was added to RAW264.7 and HepG2 cell pellets individually. The total proteins of each cell line were initially extracted from the lysates of 4×10^5^ cells. And equal amounts of the protein samples were subjected to 20% trichloroacetic acid (TCA) precipitation for 15 min. The samples were centrifuged at 15,000 g for 5 min at 4°C to remove debris. The supernatants were purified and detected usingby the method described by Drusch et al. [26], which uses an RP-HPLC method with an ortho-phthaldialdehyde (OPA)-derivatisation and fluorescence detection suited for automation. The HPLC analysis method used a 250 mm×4.6 mm C18 column (GROM-SIL 100 ODS-0 AB, Herrenberg, Germany) and detected the fluorescence excitation at 340 nm and emission at 455 nm. The peak identification of CML was confirmed by the retention time and standard addition and was quantified using an external standard. The amount of CML was calculated and expressed as a percentage.

### Intravenous administration study

After adjusting the animal board position, 0.15 mL of an ^18^F-CML solution was administered intravenously via the tail vein. The solution of ^18^F-CML was prepared just prior to the intravenous administration. The dose of ^18^F-CML was adjusted to approximately 80 µCi per mouse (n = 4), and the pH of the solution was adjusted to 7.4. Here, the specific activity corresponded to 3 MBq/nmol. Dynamic PET images were acquired continuously for 2 hours.

### Intragastric administration study

Male ICR mice weighing 18–20 g (7–8 wks old, n = 4) were kept fasting for 14 h before the end of the experiment and had free access to water before the experiments that followed. For the experiment, the mice were anesthetized and unconsciousness was maintained with a mixture of 1.5% isoflurane and nitrous oxide∶oxygen (7∶3). Approximately 3.0 MBq (80 µCi) of ^18^F-labeled CML was administered via the gastrointestinal route. Five-minute static PET images were acquired at 15, 30, 60, 120 and 150 min post-injection.

### Biodistribution studies

The biodistribution of ^18^F-CML was studied in eighteen young male ICR mice with a weight range of 18–20 g. Nine mice were used for intravenous administration and the other nine mice were used for intragastric administration.

For intravenous administration, ^18^F-CML was injected into a lateral tail vein of the animals under light ether anesthesia. The injection volume (2–3 MBq; radiochemical purity 96%) was 0.1 mL. For intragastric administration, 1.5 MBq of ^18^F-labeled CML was given via gastrointestinal tract tract.

The mice were sacrificed at 5, 60, and 120 min after administration of ^18^F-CML (three mice at each time point). The animals were sacrificed by heart puncture under ether anaesthesia. The tissues and organs of interest were isolated and counted in a well-type γ counter. Additionally, 200 µL of blood was taken from the carotid artery. The distributions of ^18^F-CML in different tissues and organs were calculated and expressed as the percent uptake of the injection dose per whole tissue (% ID).

### PET data acquisition and reconstruction

Dynamic PET studies were performed using a dedicated microPET scanner for small animals. For imaging studies, the animals were under isoflurane anesthesia and then positioned and immobilized supine with their medial axis parallel to the axial axis of the scanner with the thorax and abdominal region (organs of interest: heart, liver, and kidneys) in the center of the field of view.

Approximately 5 min was needed to set the position of the animals after intragastric or intravenous administration of the drug solution containing ^18^F-CML. Then, the emission data were acquired for 120 min. An attenuation map was created for each mouse by intravenously administering the residual ^18^F-CML after acquisition of the emission data. The acquired data were sorted into dynamic sinograms. Three-dimensional dynamic images were reconstructed by the filtered back-projection method using a Ramp filter cutoff at the Nyquist frequency with attenuation correction. The volumes of interest (VOIs) were placed on the heart, liver and kidneys. The quantitative analyses of the hepatic and renal distributions of ^18^F-CML were performed using ASIPro installed in the PET system.

### Statistical analysis

The data were expressed as the means ± SD and analyzed using SPSS v.13.0 software. For comparisons between two variables, the unpaired Student's t test was used. A two-tailed *P*<0.05 was considered statistically significant.

## Results

### Standard products ^19^F-CML synthesis

**Figure 3:**
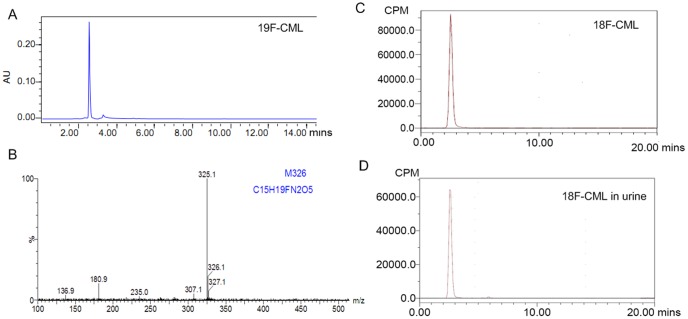
^19^F-CML/^18^F-CML identification result. A. HPLC analysis result of the standard cold product ^19^F-CML. B. LC/MS analysis result of ^19^F-CML. C. HPLC analysis of the ^18^F-CML with radioactivity detection showing radiochemical a purity of ^18^F-CML >95%. The chromatograms of the purified ^18^F-CML were characterized using a radioactivity detector, and ^19^F-CML was characterized using a UV detector (254 nm) under the same analytical HPLC conditions. The resulting ^18^F-CML was determined to have a radiochemical purity of 96% and the same retention time as ^19^F-CML. D. Radio HPLC analysis at 40 min after injection of ^18^F-CML. No metabolic products were observed in the urine.

The compound ^19^F-CML was separated using semi-preparative HPLC of the collected fractions (t_R_ = 2.6 min, Figure 3A). The identical mass peaks were observed at 325.1 m/z ([M-H]), which were indicative of the ^19^F-CML as shown in Figure 3B

### ^18^F-CML synthesis

The conjugation of CML to ^18^F-SFB occurred on a free amino group in the compound shown in Figure 1. The resulting conjugation products were purified using a reversed-phase HPLC system. Radiochemical and chemical purities of greater than 96% and a special radioactivity of 9±2 GBq/µmol were achived. The purity and identity of the products were confirmed using HPLC. [^18^F]fluorobenzoylated CML was obtained with a radiochemical purity of 96%. Starting from^18^F-SFB, the radiolabeled product could be synthesized within 60 min, including the HPLC purification. The consistent retention time between ^18^F-CML (Figure 3C) and ^19^F-CML (Figure 3A) eluted in the same HPLC system proved that the structures of the two compounds were identical.

### In vitro stability

^18^F-CML did not decompose during its synthesis and formulation. The chromatographic analyses performed showed that the complex prepared under optimal conditions had excellent stability, retaining a radiochemical purity (RCP) of 98.2±0.3% for 6 h at room temperature (26±2°C).

### In vivo stability

To better understand the relationship between the measured amount of radioactivity in the blood and urine and the amount of radiochemical compound present in the samples, it was necessary to analyze the radioactive species in blood and in urine using RP-HPLC to obtain data concerning the potential metabolization of the injected ^18^F-CML compound. The analysis showed that ^18^F-CML was stable, with more than 98% of the radioactivity in an unchanged form in the plasma and urine at 60 min after injection (Figure 3D).

### Cell uptake

**Figure 4:**
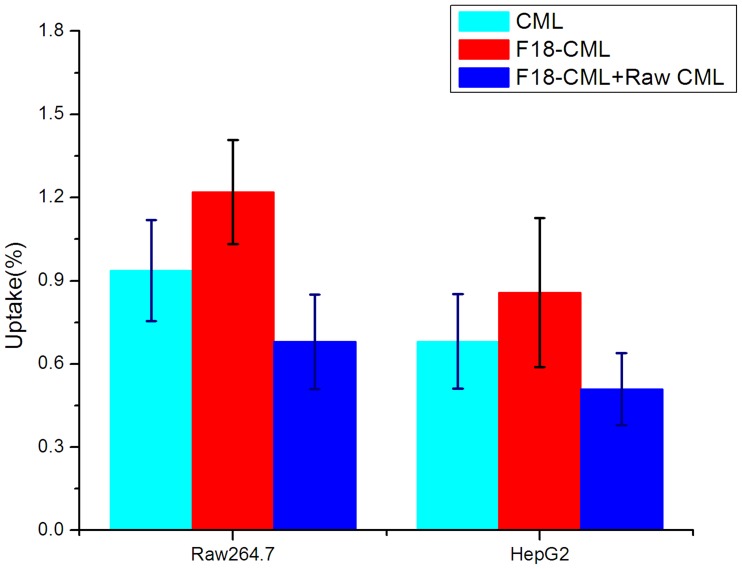
Cell uptake of ^18^F-CML and native CML. Comparison of the uptake ratios of CML (treated with 10 µg/mL native CML for 2 hours), ^18^F-CML no blocking treated, and ^18^F-CML blocked with 20 µg/ml non-radioactive compound CML in the RAW264.7 and HepG2 cell lines at 37°C.

The cell uptake of ^18^F-CML and unaltered CML was evaluated in HepG2 liver cells and RAW264.7 macrophages. The binding specificity of ^18^F-CML was evaluated using unlabelled CML-treated cells. After incubation with ^18^F-CML, the uptake of radioactivity in the untreated RAW264.7 cells (n = 3) was 1.8-fold higher than in the treated cells (*P*<0.01). The uptake of radioactivity in the untreated HepG2 cells (n = 3) was 1.6-fold higher than in the treated cells (*P*<0.01). During the first 15 min of incubation, ^18^F-CML was rapidly taken up by the HepG2 cells. After 15 min, the uptake slowed and finally reached a plateau. The 60-min cell uptake results are shown in Figure 4. Interestingly, after incubation with CML for 2 hours, the uptake ratio of unaltered CML in the untreated RAW264.7 cells (n = 3, 8.2 µg/mg of cell protein) and HepG2 cells (n = 3, 6.3 µg/mg of cell protein) was 0.3% and 0.2% less than ^18^F-CML (*P*<0.01, Figure 4). They are both phagocytosed by RAW264.7 and HepG2 cells that co-express the RAGE and Scavenger receptors, which are mediated by the SR-A [27], CD36 [28] and SR-BI [29], [30] receptors.

### Biodistribution study

Table 1 shows the distribution of ^18^F radioactivity (decay-corrected) in ICR mice after intravenous injection and intragastric administration. Intravenous administration biodistribution studies (Table 1, left) indicated that the uptake of ^18^F-CML in the kidneys was the highest among the non-target organs, which reached 29.30% ID at 5 min, then increased to 35.6% ID at 60 min. At 2 h, there was still an intensity accumulation of only 18.1% ID. In contrast, the excretion of radioactivity in the urine also occurred quite quickly. At 5 min postinjection, approximately 23.5% of the injected dose could be found in the urine. The portion of radioactivity that was delivered to the urine at 60 min post-injection was approximately 45% ID. Furthermore, minor accumulation of radioactivity was observed in the stomach, lungs, liver and intestines. However, taken together, these organs comprised less than 10% of the total injected dose. Therefore, at 60 min post-injection less than 50% of the injected dose could be found in the urine and above-mentioned organs. The remaining radioactivity was located in the circulating blood (approximately 8%) and, to a large extent, residually in the muscle tissue of the animal.

**Table 1:** Biodistribution of ^18^F-CML in different organs of ICR mice.

Organ/Uptake	Intravenous administration (%ID)			Intragastric administration (%ID)		
	5 min	60 min	120 min	5 min	60 min	120 min
Brain	3.04±0.02	0.38±0.01	0.07±0.00	1.04±0.02	0.31±0.03	0.02±0.01
Intestines	1.02±0.05	2.35±0.01	0.5±0.00	9.91±1.36	52.29±8.71	48.32±5.2
Stomach	0.35±0.02	0.02±0.01	0.01±0.00	76.85±7.01	29.73±3.91	17.87±2.1
Bladder	26.50±1.70	41.28±3.50	72.30±5.20	5.20±1.68	12.21±1.62	29.59±3.7
Lungs	1.65±0.05	1.12±0.02	0.25±0.00	0.35±0.19	0.51±0.11	0.23±0.05
Liver	17.28±2.04	9.72±1.62	3.28±0.05	1.17±0.45	1.0±0.12	0.29±0.05
Pancreas	0.9±0.04	0.01±0.0	0.00±0.02	0.2±0.01	0.32±0.02	0.05±0.00
Muscles	8.02±0.82	5.17±1.12	3.02±0.82	1.76±0.53	2.16±1.82	1.11±1.78
Kidneys	29.30±2.05	35.6±4.06	18.1±2.65	2.20±0.95	3.31±1.91	2.1±0.29
Heart	11.15±0.05	5.05±0.01	1.03±0.00	1.05±0.25	0.75±0.52	0.29±0.59

Radioactivity accumulation was expressed as the percentage of the injected dose per whole tissue (% ID) after a single intravenous injection or a single intragastric administration of 2 MBq of ^18^F-CML in 0.5 mL of saline with 2% ethanol at 3 different time points (n = 3/group, mean ± SD).

For the intragastric administration route, the level of ^18^F-CML uptake in organs is shown in Table 1, right. The uptake of ^18^F-CML in the heart, liver, kidneys and skeletal muscles was relatively lower than the uptake after intravenous administration. At 5 min, the radioactivity intensity accumulation could be measured in the stomach, while the accumulation in the intestines was very low. At 60 min, the accumulation of radioactivity was located mainly in the gastrointestinal tract, which accounted for approximately 82% ID. The remaining fraction was mainly excreted in the urine, which accounted for 12% ID. At 120 min after intragastric injection, low amounts of radioactivity could be found in the liver, kidneys and bladder. The radioactivity intensity accumulation could be measured in the intestines, while the accumulation in the stomach was relatively low. The blood uptake was only 0.95±0.09% ID percentage of wet tissue.

### Intravenous administration PET data acquisition and imaging studies

**Figure 5:**
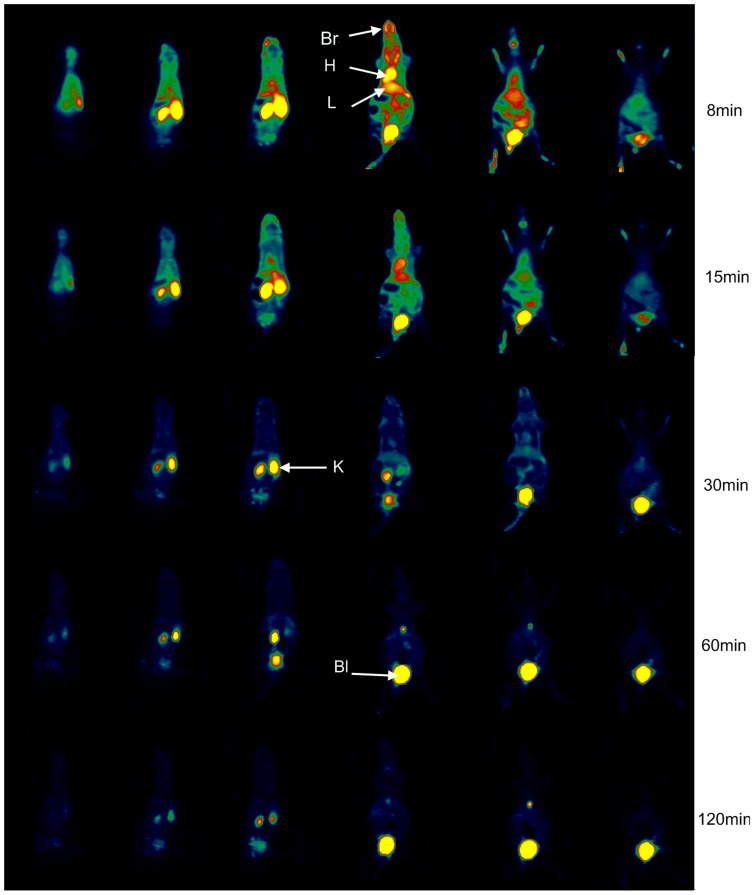
PET imaging results by intravenous administration of ^18^F-CML. Representative serial coronal whole-body dynamic PET images obtained from dynamic small animal PET scans showing the [^18^F]-radioactivity distribution at different times. From left to right horizontally, the mouse is shown in consecutive slices from dorsum to belly; from up to down vertically, the mouse is shown in slices at 8, 15, 30, 60 and 120 min after injection of ^18^F-CML. Colours indicating the measured dose of radioactivity range from black (no activity) through green, red and yellow (highest activity). H, heart; L, liver; Bl, bladder; K, kidney; Br, brain.

Figure 5 shows representative coronal PET images of the whole body at different time points after intravenous injection of ^18^F-CML. The PET study provided additional information concerning the dynamics of both the blood clearance and the organ-specific uptake of ^18^F-CML during the whole study period of 120 min. And the PET studies were in agreement with the findings of the biodistribution studies.

**Figure 6:**
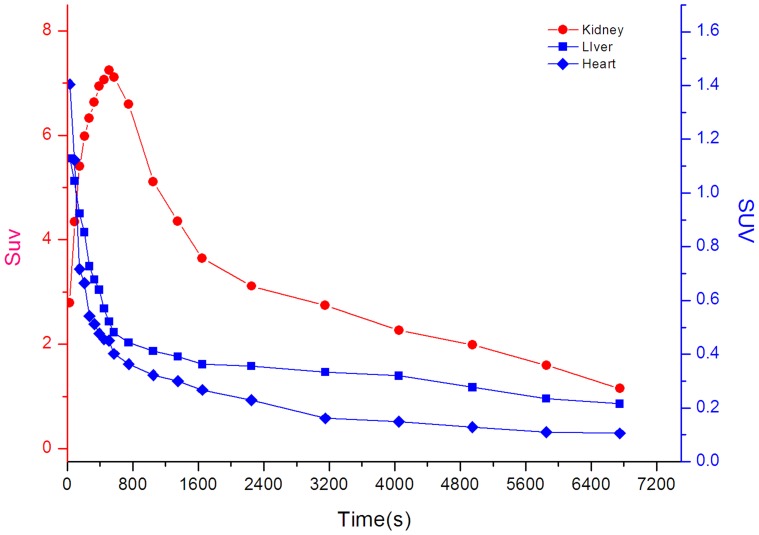
Representative time-activity double-Y axis curves of major organs (kidneys, liver and heart) derived from 120-min dynamic PET scans after intravenous administration of ^18^F-CML tracers. The kinetics of the radioactivity were calculated from a region-of-interest analysis of the dynamic small animal PET scans over the heart (diamond; mainly representing the cardiac blood pool), kidney (circles) and liver (squares). The left-Y axis data represent the kidneys, and the right-Y axis data represent the liver and heart.

After the injection of ^18^F-CML, the radioactivity was distributed throughout the whole body via the bloodstream. Therefore, most of the radioactivity could be found in the heart, which is a well-perfused organ, in the first minutes of the PET study. This is illustrated in Figure 6, which shows time–activity curves for the heart and kidneys after the injection of ^18^F-CML. As a result of perfusion, the liver could also be observed in the PET image in the first few minutes after injection. Moreover, already a few seconds after injection an accumulation of radioactivity in the kidneys could already be observed. This accumulation continued for approximately 60 min. Radioactivity accumulation in the urinary bladder represented rapid and significant excretion in urine. Most of the radioactivity was rapidly cleared from the kidneys and directly excreted via the bladder. From 60 min post-injection onwards, no selective accumulation in specific regions except the kidneys were found, and almost all of the radioactivity could be found in the bladder.

### Intragastric injection administration PET data acquisition and imaging studies

**Figure 7:**
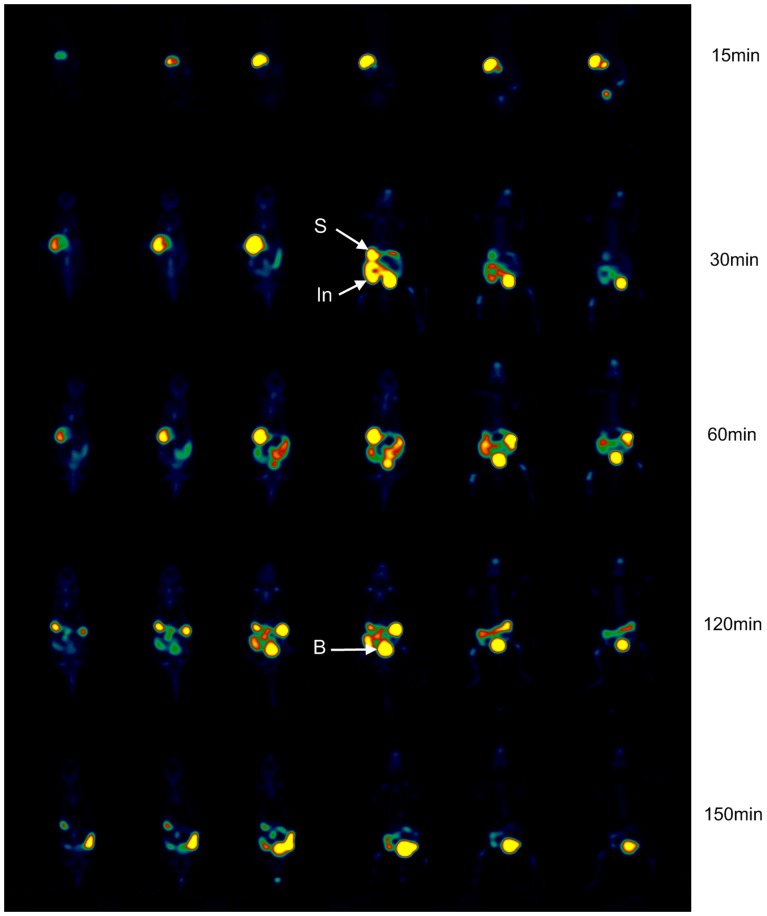
PET imaging results following intragastric administration of ^18^F-CML. Representative serial coronal whole-body dynamic PET images obtained from dynamic small animal PET scans showing the [^18^F]-radioactivity distribution at different times. From left to right horizontally, the mouse is shown in consecutive slices from dorsum to belly. From up to down vertically, the mouse is shown in slices at 15, 30, 60, 120 and 150 min after intragastric injection of ^18^F-CML. Colours indicating the measured dose of radioactivity range from black (no activity) through green, red and yellow (highest activity). S, stomach; In, Intestine; B, bladder.

After the application of ^18^F-CML via a stomach tube, the radioactivity was completely localized in the stomach for the first 15 min after administration. Subsequently, the passage of the radioactivity into the small intestine was observed. However, resorption of radioactivity from the small intestine was not observed within 2 h after application. At 150 min post intragastric injection, an intensive accumulation of radioactivity in the intestine was still observed. Much lower amounts of radioactivity in the liver and kidneys could be found. And the small amount of ^18^F-CML that was absorbed was mostly rapidly excreted in the urine at the beginning of the intragastric injection. To illustrate the gastrointestinal behaviour of the compound, a corresponding PET image is shown in Figure 7.

## Discussion

In the present study, we synthesized ^18^F-CML and found that it is a stable small molecule, and it has a decay time that is sufficiently long to obtain images. We chose ^18^F-SFB as an agent to modify CML because the fluorobenzoyl moiety acting as a surrogate for the second amino acid of a dipeptide [23]. Our study demonstrated that PET is highly capable of visualizing and kinetically analyzing the gastrointestinal absorption and intravenous biodistribution of CML-peptide as an in vivo model of low molecular weight (LMW) AGEs. To the best of our knowledge, we are the first to report the metabolism and biodistribution of peptide-bound AGEs in vivo after intragastric and intravenous administration, which significantly expands our understanding of the absorption of biological dietary AGEs and introduces practical approaches for the in vivo study of metabolism of dietary AGEs.

Interestingly, as observed in the data (Figure 5), small animal PET imaging studies showed that the ^18^F radioactivity after intravenous administration of ^18^F-CML was very rapidly cleared from the blood, entirely due to the high levels of uptake in the kidneys and liver followed by substantial renal excretion. There has been a controversy concerning which organ AGEs are mainly metabolized in [31]. Some studies suggested in the liver [32], [33], while more and more evidence supportted in the kidneys [7], [34]. Our experiment results showed that the liver was not the major site of metabolism, but rather the kidneys are the most important organs responsible for the metabolism of AGEs.

In contrast, after intragastric administration, our results indicated that ^18^F-CML was hardly absorbed by the gastrointestinal tract, which was consistent with former studies giving the conclusion that only 10% of the oral load of AGEs was intestinally absorbed and transported to the blood stream. In addition, the absorbed AGEs was fast eliminated in the urine [35]. Another reported less than 3% of the orally supplied Amadori product underlies renal excretion without further metabolism, whereas the major part of Amadori products was not resorbed during digestion [36]. Additional support for limited CML gastrointestinal bioavailability come from a study of the trans-epithelial flux of Maillard reaction products in Caco-2 intestinal epithelial cell monolayer [37], It reported that CML dipeptide transport across the intestinal epithelium is low and occurs via simple diffusion. Currently, there has been some debate over whether dietary AGEs contribute to the blood pool of AGEs in vivo. Some studies have reported that CML-rich diets significantly increase circulatory and tissue CML levels [8], [38], [39]. However, other studies have reported that dietary advanced glycation end-products consumption is independently of circulating AGE (CML) levels [7], [11], [40]. Accurate information concerning the metabolism and distribution of AGEs in vivo is lacking. Our results showed a relatively accurate dynamic whole-body distribution within 2.5 hours.

Several studies have focused on understanding the absorption, metabolism and excretion of dietary AGEs [41], [42]. These studies showed that CML is not actively transported across the epithelial monolayer which is in agree with our experimental results. However, the detailed mechanisms of intestinal absorption of AGEs in vivo are still unclear. More studies on this area are needed to understand the impact of dietary AGEs on health and aging. Our study appears to be the first report in which PET has been applied to the study of gastrointestinal absorption of dietary AGEs, which can be used as a model of LMW AGEs [23], [24]. It may be helpful to understand the potential link between the ingestion of individual dietary AGEs to evaluate their health risk. More research should be performed on other AGE models such as pyrraline and pentosidine peptides.

CML is one of the smallest AGE modifications, but it might be functionally relevant. After ^18^F-fluorobenzoylated modification of CML, although it leads to a change in charge, the formerly positively charged lysine residues still carry a negatively charged carboxylic group [43]. CML-peptides are major AGE proteins that are recognized by scavenger receptors involved in the recognition and catabolism of AGEs in vivo [21]. Although ^18^F-CML may be different from free CML physically, they showed some similar biocharacteristics in our in vitro experiments. Perhaps the following explanation holds true: AGE receptor recognition occurs primarily through the binding of negatively charged regions of AGE-modified proteins rather than interaction with distinct glycation moieties on the amino acid side chains of proteins [44], [45], [46].

The limitation of our study is the lack of experiments on pathology in animal models. The relative short half-lives of the radioisotopes applied in PET compared with the rate of digestive processes might limit the application of this approach. Because it is almost impossible to achieve the same formulation used by drug manufacturers within the timeframe of the short half-lives of PET radionuclides, these types of studies are extremely difficult to perform. Rapin et al. [47] suggested that normally dietary AGEs cross the intestinal wall poorly. However the elimination of AGEs might be very different if intestinal permeability (IP) is elevated by food allergies [48], and AGEs can affect the IP itself [49]. Inefficient clearance of AGE-rich peptides and recirculation of these ‘toxic’ molecules might be responsible for vascular damage in diabetic patients. Recent studies have shown that in patients with chronic renal failure (CRF), reduced renal metabolism of AGEs likely accounts for the accumulation of AGEs in the serum [5], [50]. Further experiments with various pathological conditions are needed to establish whether AGE-rich diets can cause an accumulation of AGEs that is harmful to animal or human bodies.

## Conclusion

In this study, we successfully coupled CML with the positron-emitting radionuclide ^18^F through the prosthetic labeling group ^18^F-SFB. In vivo pharmacokinetics showed that ^18^F-CML was relatively metabolically stable. Dynamic scans demonstrated that PET is a prominent tool for evaluating AGE-peptide metabolites in vivo following both intravenous and intragastric administration. Through our experiments, we observed that ^18^F-CML-peptides were largely not absorbed from the gastrointestinal tract, and were cleared rapidly by the kidneys after intravenous administration. The elimination rate via the kidneys was faster than the gastrointestinal absorption rate. Thus we concluded that exogenous CML-peptides from dietary sources likely could not accumulate in healthy bodies. Further study should be undertaken to investigate this model under pathological gastrointestinal conditions such as enteritidis and gastrointestinal tract allergies, and under pathological kidney conditions such as diabetic nephropathy.
